# Calcium Channel Ca_V_2.3 Subunits Regulate Hepatic Glucose Production by Modulating Leptin-Induced Excitation of Arcuate Pro-opiomelanocortin Neurons

**DOI:** 10.1016/j.celrep.2018.09.024

**Published:** 2018-10-09

**Authors:** Mark A. Smith, Loukia Katsouri, Samuel Virtue, Agharul I. Choudhury, Antonio Vidal-Puig, Michael L.J. Ashford, Dominic J. Withers

**Affiliations:** 1 Metabolic Signalling Group, MRC London Institute of Medical Sciences, London W12 0NN, UK; 2 Metabolic Research Laboratories, Institute of Metabolic Science, University of Cambridge, Cambridge CB2 0QQ, UK; 3 Division of Molecular and Clinical Medicine, Ninewells Hospital and Medical School, University of Dundee, Dundee DD1 9SY, UK; 4 Institute of Clinical Sciences, Imperial College London, Du Cane Road, London W12 0NN, UK

**Keywords:** leptin, POMC neuron, glucose, liver, insulin resistance, calcium channel, hypothalamus, diabetes

## Abstract

Leptin acts on hypothalamic pro-opiomelanocortin (POMC) neurons to regulate glucose homeostasis, but the precise mechanisms remain unclear. Here, we demonstrate that leptin-induced depolarization of POMC neurons is associated with the augmentation of a voltage-gated calcium (Ca_V_) conductance with the properties of the “R-type” channel. Knockdown of the pore-forming subunit of the R-type (Ca_V_2.3 or *Cacna1e*) conductance in hypothalamic POMC neurons prevented sustained leptin-induced depolarization. *In vivo* POMC-specific *Cacna1e* knockdown increased hepatic glucose production and insulin resistance, while body weight, feeding, or leptin-induced suppression of food intake were not changed. These findings link *Cacna1e* function to leptin-mediated POMC neuron excitability and glucose homeostasis and may provide a target for the treatment of diabetes.

## Highlights

• Leptin activates an R-type calcium channel in POMC neurons
• Knockdown of pore subunit *Cacna1e* blocks sustained POMC depolarization by leptin
• Mice with POMC neuron knockdown of *Cacna1e* have increased hepatic glucose production
• *Cacna1e* plays a role in leptin regulation of glucose homeostasis by POMC neurons

## Graphical Abstract

**Figure:**
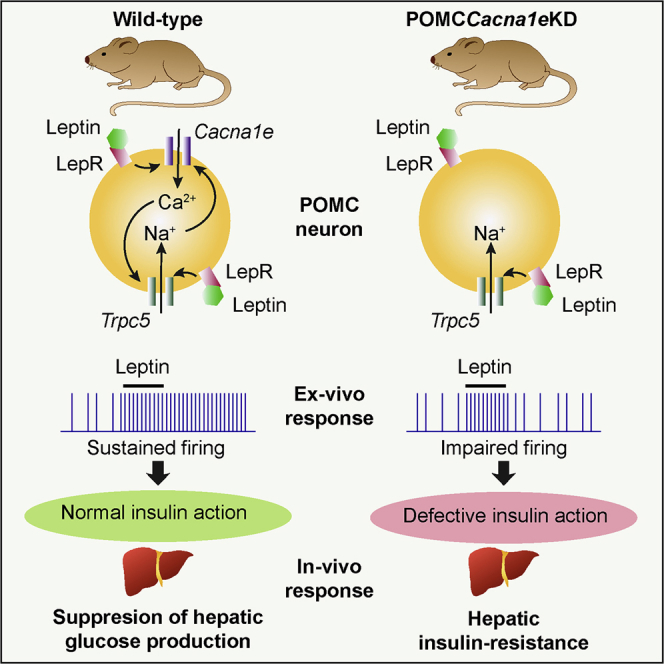

## Teaser

Smith et al. show that an R-type calcium channel containing the pore subunit *Cacna1e* mediates long-term depolarization of hypothalamic POMC neurons by leptin. Mice with POMC neuron knockdown of *Cacna1e* display elevated hepatic glucose production, demonstrating that leptin utilizes *Cacna1e* in POMC neurons to regulate glucose homeostasis.

## Introduction

The CNS regulates glucose homeostasis by modulation of peripheral insulin sensitivity and hepatic glucose production (HGP) (Ruud et al., 2017, Schwartz et al., 2013). Insulin and leptin play a key role acting on hypothalamic arcuate nucleus agouti-related peptide (AgRP) and pro-opiomelanocortin (POMC)-expressing neurons to regulate glucose homeostasis (Ruud et al., 2017, Schwartz et al., 2013). Insulin signaling in AgRP neurons chronically modulates glucose metabolism by altering HGP (Könner et al., 2007). Leptin signaling in POMC neurons chronically regulates insulin sensitivity and HGP (Berglund et al., 2012). Leptin administered to the mediobasal hypothalamus (MBH) has an anti-diabetic effect (Morton and Schwartz, 2011) and re-expression of leptin receptors (LepRs) into POMC neurons of LepR-deficient mice restores impaired glucose homeostasis (Berglund et al., 2012). However, the precise mechanisms by which leptin signaling regulates POMC neuron activity and thereby controls glucose metabolism are not fully understood.

Leptin increases POMC neuron excitability in a phosphatidylinositol-3 kinase (PI3K)-dependent manner, and prenatal loss of POMC PI3K signaling impairs insulin sensitivity and increases HGP (Al-Qassab et al., 2009, Hill et al., 2008, Shi et al., 2013). Activation of a non-selective cation conductance is thought to underlie POMC excitation (Cowley et al., 2001, Hill et al., 2008). Qiu et al. (2010) showed that leptin excited POMC neurons by activation of a channel composed of transient receptor potential-C (Trpc) 1, 4, 5, and 7 subunits, an action potentiated by raised intracellular calcium. Furthermore, *Trpc5* knockout in mouse POMC neurons reduced resting excitability, abolished leptin excitation and feeding suppression, increased body weight, and lowered energy expenditure with no effect on glucose homeostasis (Gao et al., 2017).

Here, we show that leptin modulates a Ca_V_2.3 (*Cacna1e*) subunit containing voltage-dependent calcium channel (Ca_V_), which is required for chronic leptin-induced excitation of POMC neurons. Knockdown of *Cacna1e* in arcuate POMC neurons impairs regulation of HGP in adult mice.

## Results

### Identification of a Ca_V_ Conductance Modulated by Leptin in POMC Neurons

**Figure 1:**
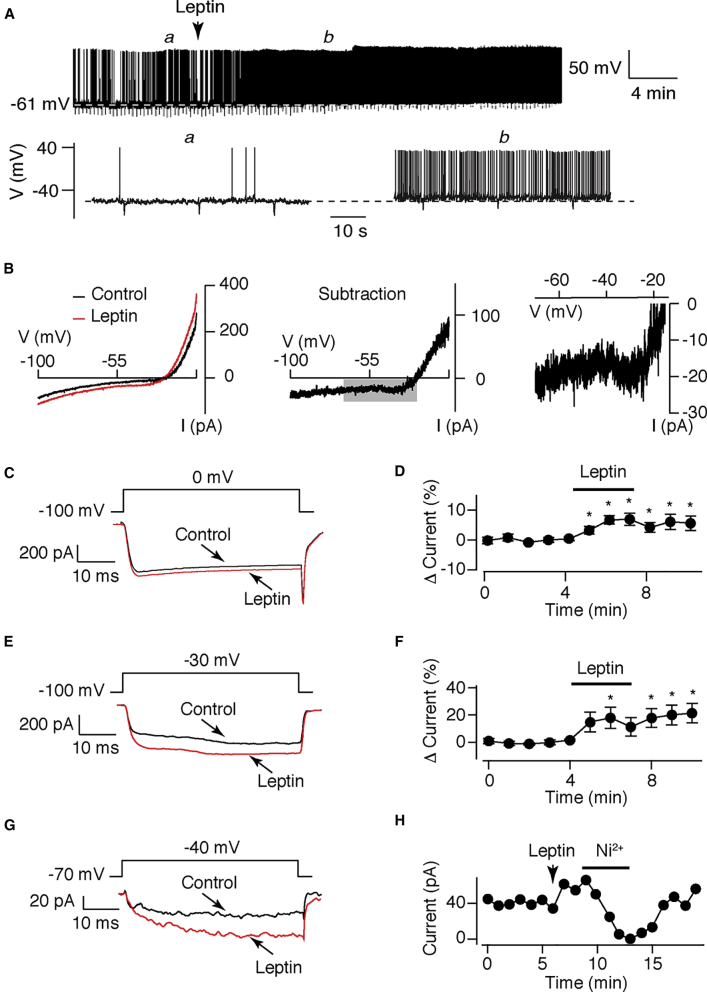
Leptin Augments a Voltage-Gated Calcium Conductance (A) Representative continuous whole-cell current-clamp trace (upper) and expanded sections (lower, as indicated by italic letters) from a POMC neuron recorded in intact slices. Locally applied leptin (50 nM; 2 min where indicated) depolarized and increased firing frequency of a sub-population of POMC neurons. (B) Representative currents evoked by hyperpolarizing voltage ramps (−10 to −100 mV; 2-s duration) in the presence and absence of leptin (50 nM), where indicated (left). Currents evoked by leptin (subtraction of control from leptin currents) is shown for the whole current-voltage relationship (middle) with the switch to inward current (boxed region) shown expanded (right). (C) Barium currents evoked from POMC neurons in intact slices by voltage steps to 0 mV from a holding potential of −100 mV. Mean (five consecutive test pulses) ensemble barium currents are shown immediately before (black) and following (red) locally applied leptin (50 nM), where indicated. (D) Diary plots of barium current amplitude evoked at 0 mV plotted against time in the absence and presence of leptin (mean ± SEM; n = 10). One-way ANOVA (F(12,101) = 2.51; p = 0.006) with deviation from baseline control examined by one-sample t test. ^∗^p < 0.05. (E) Barium currents evoked in intact slices by voltage steps to −30 mV from a holding potential of −100 mV. Mean ensemble barium currents before (black) and following (red) locally applied leptin (50 nM), where indicated. (F) Diary plots of barium current amplitude evoked at −30 mV plotted against time before and after leptin (50 nM) application (mean ± SEM, n = 10). One-way ANOVA: absence (F(12–119) = 2.91; p = 0.0016) with deviation from baseline control examined by one-sample t test. ^∗^p < 0.05. (G) Barium currents were evoked from dispersed POMC neurons by voltage steps to −40 mV from a holding potential of −70 mV in the absence (black) and presence of leptin (red). (H) Diary plot for the recording shown in (J) and following addition and removal of bath applied NiCl_2_ (100 μM, where indicted).

As shown previously (Al-Qassab et al., 2009, Choudhury et al., 2005, Claret et al., 2007, Cowley et al., 2001, Hill et al., 2008, Qiu et al., 2010, Smith et al., 2015, Williams et al., 2010), local application of leptin (50 nM; 2 min) caused long-lasting (>1-hr) depolarization (n = 33; paired t test, t(32) = 3.63; p < 0.001; Figure 1A) in a sub-population of POMC neurons consistent with recent RNA-sequencing (RNA-seq) data showing that only ∼50% of POMC neurons contain the LepR (Lam et al., 2017). Cells that did depolarize to leptin (ΔVm, +4.4 ± 1.0 mV; n = 15 responsive cells out of 33) had an increased firing frequency (2.9 ± 0.8 to 4.2 ± 0.9 Hz; n = 15 responsive cells out of 33) and reduced input resistance (87.2 ± 7.7% of control; n = 15 responsive cells out of 33). In voltage-clamp recordings, leptin increased the holding current by −11.4 ± 4.2 pA (n = 8; paired t test, t(7) = 2.70; p < 0.05) at −70 mV. In hyperpolarizing voltage-ramps (−10 to −100 mV) without ion channel pharmacology, leptin evoked an outwardly rectifying current that reversed at −32.2 ± 3.6 mV (n = 8; Figure 1B). While these properties are consistent with the activation of a Trpc5 subunit-containing conductance, an additional leptin-dependent inward current was evoked at potentials greater than −40 mV (Figure 1B). Given the dependence of the Trpc5 conductance on intracellular calcium, we therefore hypothesized that leptin may also activate a voltage-gated calcium (Ca_V_) conductance.

To investigate the identity and properties of POMC Ca_V_ currents, whole-cell recordings were performed with a CsCl (130 mM) internal solution and extracellular divalent cations replaced with 10 mM BaCl_2_, to block K^+^ channels and enhance Ca_V_ currents. Recordings were also performed with tetrodotoxin and inhibitors of fast synaptic transmission. POMC neurons were voltage clamped at –70 mV and Ba^2+^ currents evoked by 50-ms voltage steps from –100 to 0 mV (Figure S1A). We observed run-up in current amplitude over 5 min to a stable level (Figure S1B) with the current completely inhibited by CdCl_2_ (100 μM; Figure S1A). Ca_V_ are classified as L, T, P, Q, N, and R types based on molecular, biophysical, and pharmacological properties (Catterall et al., 2005). Thus, a pharmacological dissection of POMC Ca_V_ was performed using Ba^2+^ currents at 0 mV. Addition of mibefradil (10 μM), an R- and T-type (and partial L-type) inhibitor, followed by the N-type blocker ω-conotoxin-GVIA (200 nM) and L-type blocker nimodipine (10 μM), each inhibited evoked currents by similar magnitudes (Figures S1C and S1E). In the presence of all three Ca_V_ blockers, application of the P- and Q-type blocker ω-agatoxin-IVA (200 nM) reduced current amplitude further (Figures S1D and S1E). This cocktail of blockers did not completely suppress POMC Ca_V_ currents, indicating the presence of a resistant (“R-type”) conductance. However, 50 nM SNX-484 (which blocks R currents in most but not all cells; Newcomb et al., 1998) did not affect the Ba^2+^ current amplitude (Figure S1F).

Next, we tested whether leptin modulates a specific Ca_V_ current. Following establishment of a stable current amplitude, leptin increased the Ba^2+^ current at 0 mV (Δ current, −27.5 ± 9.8 pA; n = 10; paired t test, t(9) = 2.81; p < 0.03) and by a greater magnitude at −30 mV (Δ current, –79.7 ± 30.4 pA; n = 10; paired t test, t(9) = 2.63; p < 0.03), which was sustained following leptin removal (Figures 1C–1F). Augmentation of the Ba^2+^ current was unaffected by 10 μM nimodipine (Δ current at 0 mV, –26.6 ± 8.6 pA; n = 10; paired t test, t(9) = 3.08; p < 0.02). However, leptin-induced augmentation was prevented by 10 μM mibefradil (Δ current at −30 mV, –8.0 ± 8.9 pA; n = 9; paired t test, t(8) = 0.90; p = 0.39) and a PI3K inhibitor TGX-221 (1 μM; Δ current, –13.2 ± 23.4 pA; n = 13; paired t test, t(12) = 0.57; p = 0.58). We next examined leptin action on Ba^2+^ currents in acutely dissociated POMC neurons to exclude the possibility that changes in network activity were responsible for the augmentation of the Ca_V_ conductance. However, the Ba^2+^ current evoked at −40 mV was increased by leptin in dispersed POMC neurons by 24.9 ± 0.9% (n = 7; paired t test, t(6) = 2.65; p < 0.04; Figure 1G) and blocked by NiCl_2_ (100 μM; n = 3; Figure 1H), which inhibits T- and R-type conductances. Although the mibefradil-sensitive Ba^2+^ current has slow inactivation kinetics, we assessed leptin action on T-type calcium channels (also Ni^2+^ and mibefradil sensitive). Ba^2+^ currents, elicited at –50 mV in dispersed POMC neurons, displayed the rapid and transient current profile of T-type channels (t_inact_, 3.1 ± 0.9 ms; n = 4; Figure S1G). Leptin did not alter the amplitude (I_max_: control, 148.1 ± 62.7 pA, versus leptin, 166.6 ± 50.7 pA; n = 4; paired t test, t(3) = 0.68; p = 0.55) or steady-state inactivation (V_0.5_: control, −91.8 ± 2.5 mV, versus leptin, −91.4 ± 24.7 mV; n = 4; paired t test, t(3) = 0.12; p = 0.91; Figures S1G and S1H) of these currents.

### Cell-Specific Knockdown of Cacna1e in POMC Neurons

**Figure 2:**
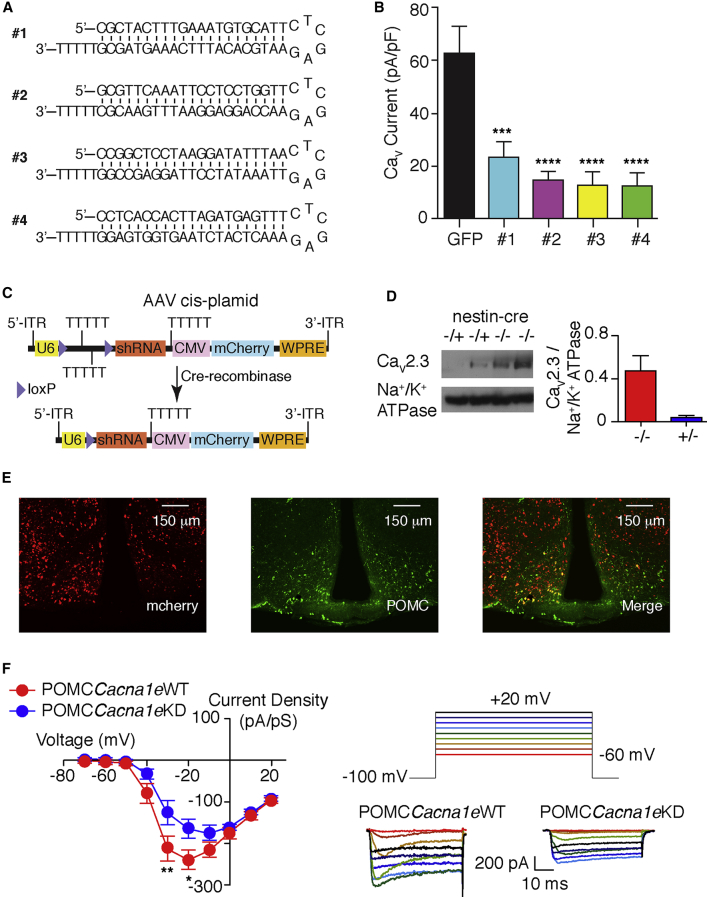
shRNA Knockdown of *Cacna1e* in POMC Neurons (A) shRNA sequences targeting the mouse *Cacna1e* gene. (B) Barium currents were evoked in GT1-7 cells by voltage steps to 0 mV from a holding potential of −100 mV. Cells were transfected with GFP or one of the four shRNA sequences (#1–4) shown in (A). Mean ± SEM; n = 9. One-way ANOVA (F(4,40) = 11.21, p < 0.0001); Bonferroni post hoc, ^∗∗∗∗^p < 0.0001. (C) Diagrammatic representation of the associated-adenoviral (AAV) expression vector before (top) and after cre recombination (bottom). (D) Western blot analysis of Ca_V_2.3 (*Cacna1e*) protein (top), Na^+^/K^+^ ATPase loading control (bottom), and quantification (right bar chart; mean ± SEM) for brain lysates from wild-type (−/−) and nestinCre (−/+) mice injected with AAV particles containing the shRNA #2 sequence shown in (A). (E) POMCCre mice injected with the shRNA construct in the MBH. Cre-independent mCherry expression (left) and POMC immunohistochemistry (middle) are shown to co-localize (right). (F) Mean current-voltage plot (left) and representative traces (right) for Ba^2+^ currents evoked in POMC neurons at voltages between −60 and +20 mV (as denoted by rainbow colors, right) in POMC*Cacna1e*WT and POMC*Cacna1e*KD mice (mean ± SEM; n = 14–27). Two-way repeated-measures (RM) ANOVA (interaction: F(1,39) = 2.82, p = 0.0032); Bonferroni post hoc, ^∗^p < 0.05, ^∗∗^p < 0.01, ^∗∗∗^p < 0.001.

These characteristics of the leptin-modulated Ca_V_ conductance are consistent with R type, where the pore-forming subunit is Ca_V_2.3 (*Cacna1e*). To investigate its role in POMC neurons, we used interference short hairpin RNA (shRNA) sequences directed toward *Cacna1e* (Figure 2A). Hypothalamic GT1-7 cells, which express R-type Ca_V_, were independently transfected with each shRNA sequence directed toward *Cacna1e* or GFP as a control and Ba^2+^ currents evoked at 0 mV. For each shRNA sequence, peak Ba^2+^ currents were reduced by 75%–85% when compared to GFP-transfected cells (Figure 2B). To target POMC cells *in vivo*, we used the approach of Hitz et al. (2007), whereby a loxP flanked stop cassette was introduced between the U6 promoter and the 5′ end of the shRNA sequence. To identify expression, mCherry driven by the CMV promoter was inserted downstream of the shRNA (#2) sequence in an associated-adenovirus (AAV) expression vector (Figure 2C). To test the efficacy of this strategy, brains of wild-type (WT) (−/−) and nestin-cre-recombinase (−/+)-expressing mice were injected with viral particles containing the shRNA sequence directed to *Cacna1e*. Ca_V_2.3 (*Cacna1e*) protein was reduced in AAV-injected brains in cre-positive mice compared to WT littermates (Figure 2D), demonstrating effective knockdown of *Cacna1e*. Viral particles were then injected into the MBH of WT and POMCCre mice to generate POMC*Cacna1e*WT and POMC*Cacna1e*KD mice, respectively. To control for potential off-target effects, we also injected a cre-dependent shRNA scramble virus into POMCCre mice to produce POMC*shRNAscramble* mice. Immunohistochemistry for POMC and mCherry confirmed expression in POMC neurons and the MBH (Figure 2E). Expression of mCherry was examined post mortem in all mice to confirm correct viral placement. For *in vitro* brain slice studies, as mCherry expression was cre independent, we crossed POMCCre with POMCGFP mice to visualize POMC neurons. Current-voltage relationships of Ba^2+^ currents were recorded from mCherry and GFP-positive arcuate neurons in POMC*Cacna1e*WT and POMC*Cacna1e*KD mice. Consistent with knockdown of R-type Ca_V_ in Cre-positive POMC neurons, peak current density was reduced with a shift in the current-voltage relationship to depolarizing potentials (Figure 2F). It is likely that the remaining barium currents are a mix of the other classes of Ca_V_ conductance present in POMC neurons (Figures S1C–S1H).

**Figure 3:**
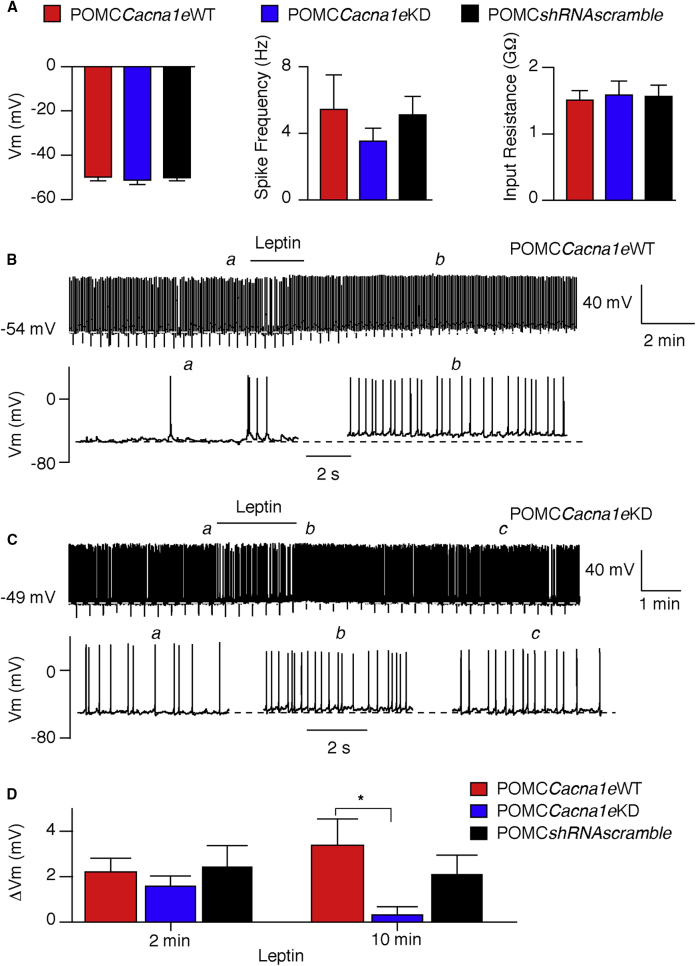
Knockdown of *Cacna1e* Prevents Chronic Leptin-Induced Depolarization (A) Bar charts showing resting membrane potential (Vm, left), spike firing frequency (middle), and input resistance (right) in POMC neurons from POMC*Cacna1e*WT (red; n = 12), POMC*Cacna1e*KD (blue; n = 15), and POMC*shRNAscramble* (black; n = 9) mice. Mean ± SEM; one-way ANOVA (Vm: F(2,33) = 0.17, p = 0.85; spike: F(2,33) = 0.61, p = 0.55; input: F(2,33) = 0.06, p = 0.94). (B and C) Representative continuous whole-cell current-clamp traces (upper) and expanded sections (lower) from POMC neurons recorded in slices from POMC*Cacna1e*WT (B) and POMC*Cacna1e*KD (C) mice. Leptin was locally applied (50 nM; 2 min), where indicated. (D) Bar charts showing leptin-induced depolarization (inclusive of non-responding cells) 2 min (left) and 10 min (right) post-leptin application in POMC neurons from POMC*Cacna1e*WT (red; n = 12), POMC*Cacna1e*KD (blue; n = 15), and POMC*shRNAscramble* (black; n = 9) mice. Mean ± SEM; one-way ANOVA (F(5,66) = 2.36; p = 0.05); Bonferroni post hoc ^∗^p < 0.05.

Resting Vm, firing frequency, and input resistance were unaltered between WT and *Cacna1e* knockdown POMC neurons (Figure 3A). During the initial 2-min application period, leptin (50 nM) equally depolarized POMC*Cacna1e*WT (n = 12; paired t test, t(12) = 3.98; p < 0.003), POMC*Cacna1e*KD (n = 15; paired t test, t(14) = 3.69; p < 0.003), and POMC*shRNAscramble* (n = 9; paired t test, t(8) = 2.59; p < 0.04) neurons (Figures 3B–3D). However, 10 min after leptin application, depolarization was only sustained in the POMC*Cacna1e*WT and POMC*shRNAscramble* neurons (Figure 3D), suggesting that Ca_V_2.3 subunit is required for the long-term depolarizing activity of leptin.

### Knockdown of *Cacna1e* Does Not Abolish Leptin Suppression of Feeding

**Figure 4:**
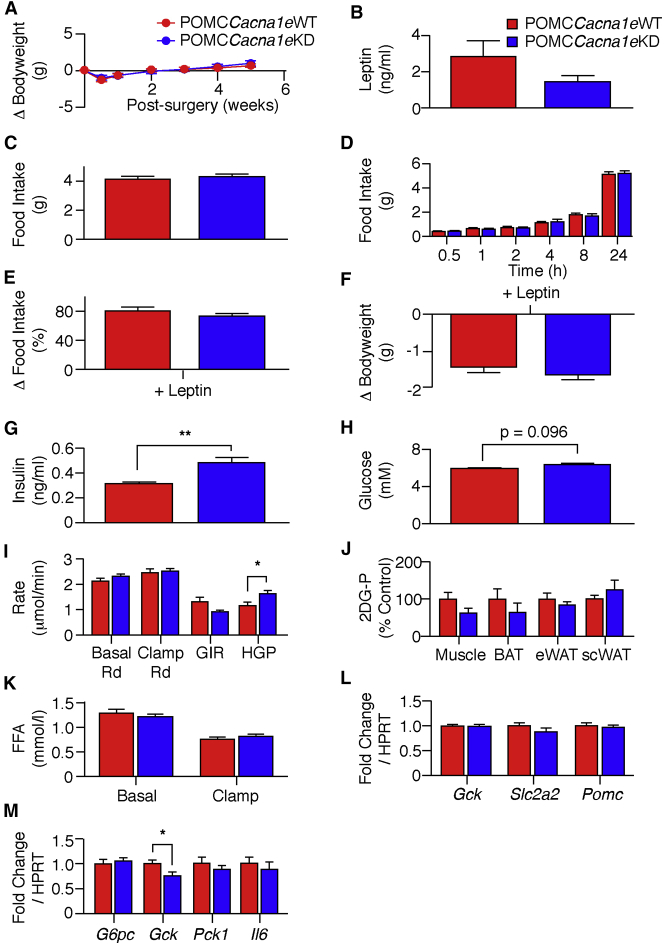
POMC-Specific Knockdown of *Cacna1e* Does Not Affect Food Intake but Causes Insulin Resistance (A) Change in body weight in 5-month-old male POMC*Cacna1e*WT (red) and POMC*Cacna1e*KD (blue) mice following stereotaxic surgery (mean ± SEM; n = 17–22). Two-way RM ANOVA (F(1,37) = 0.0112; p = 0.916). (B) Fasted serum leptin concentration in 7-month-old male POMC*Cacna1e*WT (red) and POMC*Cacna1e*KD (blue) mice (mean ± SEM; n = 11–14). Unpaired t test (t(23) = 1.62; p = 0.119). (C) Ad libitum daily food intake in 5-month-old male POMC*Cacna1e*WT (red) and POMC*Cacna1e*KD (blue) mice (mean ± SEM; n = 14–16). Unpaired t test (t(28) = 0.506; p = 0.617). (D) Cumulative 24-hr food intake following an overnight fast in 5-month-old male POMC*Cacna1e*WT (red) and POMC*Cacna1e*KD (blue) mice (mean ± SEM; n = 14–16). Two-way RM ANOVA (F(1,28) = 0.0024; p = 0.962). (E) Change in food intake following leptin administration (1.5 mg/kg twice daily for 3 days) expressed as percentage of vehicle in 6-month-old POMC*Cacna1e*WT (red) and POMC*Cacna1e*KD (blue) mice (mean ± SEM; n = 13–16). Unpaired t test (t(27) = 1.20; p = 0.240). (F) Change in body weight following leptin administration in the mice shown in (E). Unpaired t test (t(27) = 0.964; p = 0.344). Mean ± SEM. (G) Fasted serum insulin concentration in 7-month-old male POMC*Cacna1e*WT (red) and POMC*Cacna1e*KD (blue) mice (mean ± SEM; n = 21–22). Unpaired t test (t(41) = 3.51; p = 0.0011). ^∗∗^p < 0.01 (H) Fasted blood glucose concentration in 7-month-old male POMC*Cacna1e*WT (red) and POMC*Cacna1e*KD (blue) mice (mean ± SEM; n = 11–17). Unpaired t test (t(26) = 1.73; p = 0.0957). (I) Glucose disposal (Rd), glucose infusion rate (GIR), and HGP during basal and hyperinsulinemic-euglycemic clamp conditions from POMC*Cacna1e*WT (red) and POMC*Cacna1e*KD (blue) mice (mean ± SEM; n = 8). Unpaired t test: basal Rd (t(14) = 1.23; p = 0.240); clamp Rd (t(14) = 0.396; p = 0.698); GIR (t(14) = 1.90; p = 0.079); HGP (t(14) = 2.37; p = 0.0325). ^∗^p < 0.05. (J) Skeletal muscle, BAT, epidermal WAT (eWAT), and subcutaneous WAT (scWAT) uptake of ^14^C-2-deoxyglucose-phosphate (2-DG-P) in hyperinsulinemic-euglycemic clamped POMC*Cacna1e*WT (red) and POMC*Cacna1e*KD (blue) mice described in (C). Mean ± SEM. Unpaired t test: muscle (t(14) = 1.71, p = 0.110); BAT (t(12) = 0.942, p = 0.365); eWAT (t(14) = 0.917; p = 0.375); scWAT (t(10) = 0.825; p = 0.428). (K) Serum free-fatty acid (FFA) concentration during basal and hyperinsulinemic-euglycemic clamp conditions from POMC*Cacna1e*WT (red) and POMC*Cacna1e*KD (blue) mice described in (C). Mean ± SEM. Unpaired t test: basal (t(14) = 0.795; p = 0.440); clamp (t(14) = 0.990; p = 0.339). (L) qPCR for *Gck*, *Slc2a2*, *Pomc* in hypothalamic tissue from fasted POMC*Cacna1e*WT (red) and POMC*Cacna1e*KD (blue) mice (mean ± SEM; n = 13–17). Unpaired t test: *Gck* (t(27) = 0.148, p = 0.883); *Slc2a2* (t(27) = 1.15; p = 0.259); *Pomc* (t(27) = 0.417; p = 0.680). (M) Expression of *G6pc*, *Gck*, *Pck1*, and *IL6* in liver tissue from fasted POMC*Cacna1e*WT (red) and POMC*Cacna1e*KD (blue) mice (mean ± SEM; n = 12–16). Unpaired t test: *G6pc* (t(27) = 0.486, p = 0.631); *Gck* (t(25) = 2.15; p = 0.0418); *Pck1* (t(27) = 0.795; p = 0.433); IL6 (t(27) = 0.578; p = 0.568). ^∗^p < 0.05.

Prenatal leptin receptor deletion in POMC neurons increases body weight, fat mass, and serum leptin. However, knockdown of POMC neuron *Cacna1e* in adult mice did not alter body weight or fasted serum leptin compared to POMC*Cacna1e*WT littermates (Figures 4A and 4B). Ad libitum or fast-refed food intake were also unaffected (Figures 4C and 4D), and exogenous leptin suppressed feeding and reduced body weight equally in POMC*Cacna1e*WT and POMC*Cacna1e*KD mice (Figures 4E and 4F).

### Knockdown of *Cacna1e* in POMC Neurons Leads to Increased HGP and Insulin Resistance

Leptin action in the MBH regulates peripheral glucose homeostasis including HGP and insulin sensitivity, and deletion of signaling molecules in POMC neurons impairs peripheral glucose disposal and insulin sensitivity (Ruud et al., 2017). Thus, we measured fasted serum insulin and glucose levels in POMC*Cacna1e*WT, POMC*Cacna1e*KD, and POMC*shRNAscramble* littermate mice 8 weeks after viral injection. Serum insulin was increased in POMC*Cacna1e*KD mice compared to POMC*Cacna1e*WT littermates (Figure 4G) with glucose levels unaltered (Figure 4H). Similarly, serum insulin levels were increased (unpaired t test, t(8) = 3.14; p < 0.04) in POMC*Cacna1e*KD (0.74 ± 0.03 ng/mL; n = 6) mice when compared to POMC*shRNAscramble* (0.48 ± 0.10 ng/mL; n = 4) littermate controls. This suggested the presence of insulin resistance, and so we undertook hyperinsulinemic-euglycemic clamp studies. Under basal and hyperinsulinemic conditions, glucose disposal (Rd) rate was unchanged (Figure 4I). However, HGP was significantly increased with a trend (unpaired t test, t(14) = 1.90; p = 0.078) to lower glucose infusion rates (GIRs) in POMC*Cacna1e*KD mice (Figure 4I). 2-Deoxyglucose-phosphate (2DG-P) uptake into muscle, brown adipose tissue (BAT), and white adipose tissue (WAT) was unaltered between POMC*Cacna1e*WT and POMC*Cacna1e*KD mice (Figure 4J). Insulin-induced suppression of serum free-fatty acid (FFA) production was also unaffected by *Cacna1e* knockdown in POMC neurons (Figure 4K).

To investigate potential mechanism(s) for insulin resistance, we performed qPCR in hypothalamic and liver tissue from fasted mice. We observed no differences in hypothalamic expression of *Pomc*, glucokinase (*Gck*), or glucose transporter-2 (*Slc2a2*) in POMC*Cacna1e*WT and POMC*Cacna1e*KD mice (Figure 4L). However, liver *Gck* expression was reduced in POMCCacna1eKD mice, while interleukin-6 (*Il6*), phosphoenolpyruvate carboxykinase (*Pck1*), and glucose-6-phosphatase (*G6pc*) were unaltered (Figure 4M).

## Discussion

Our studies identify in POMC neurons a leptin-sensitive Ca_V_ current with R-type characteristics containing the pore-forming subunit *Cacna1e*. This plays a key role in mediating the sustained effects of leptin on POMC neuron excitability and the subsequent regulation of glucose homeostasis.

Leptin excites POMC neurons by activation of a non-selective conductance composed of Trpc subunits (Qiu et al., 2010). This activation was dependent on internal calcium, as found for *Trpc5* subunits in heterologous expression systems (Blair et al., 2009). Consistent with Trpc activation, we also observed that leptin caused a reduction in input resistance at resting potentials and increased inward current at steady-state potentials. In hyperpolarizing voltage ramps, leptin induced a current that reversed close to the predicted reversal potential for a non-selective cation channel. Recently, Gao et al. (2017) deleted *Trpc5* from POMC neurons, causing a loss of leptin-induced depolarization and reduced basal excitability. Many non-selective conductances, including *Trpc5*, are modulated by raised intracellular calcium, and since we also observed a leptin-activated voltage-dependent conductance, we postulated that this could facilitate leptin-induced POMC excitation. As *Cacna1e* channels are likely to be inactive at resting potentials (approximately –50 mV), activation of *Trpc5* and/or increased firing and excitatory synaptic input may be required to activate *Cacna1e* channels, allowing calcium entry to augment *Trpc5* channel opening. Such a positive feedforward mechanism could explain the sustained depolarization induced by brief exposure to leptin. Indeed, *Cacna1e* knockdown prevented chronic but not acute leptin-induced depolarization, suggesting that *Trpc5* activation without calcium entry is insufficient to depolarize POMC neurons over longer periods. Given leptin’s dependence on uptake mechanisms to enter the brain (Banks and Farrell, 2003), it is not surprising that circulating leptin slowly activates POMC neurons *in vivo* (Beutler et al., 2017). Thus, it is far more probable that the loss of *Cacna1e* would impact on the overall magnitude of leptin response *in vivo* rather than altering the temporal pattern of activation.

Prenatal deletion of LepR in POMC neurons does not affect food intake or energy expenditure but causes obesity and reduces *Pomc* expression (Balthasar et al., 2004). Overexpression of LepR in all POMC neurons in mice globally *null* for the LepR diminishes hyperphagia, whereas re-introduction of LepR into POMC neurons originally containing LepR minimally affects food intake (Berglund et al., 2012, Huo et al., 2009). Nevertheless, both models displayed improved glucose and insulin sensitivity. POMC neurons are important for hepatic parasympathetic nerve activity in response to leptin (Bell et al., 2018), but it is unclear whether POMC neurons regulate HGP directly through the autonomic nervous system, or indirectly by altering metabolic hormones. Recently, POMC neuron-specific deletion of the LepR in adult mice has been shown to impair insulin-induced suppression of HGP independent of changes in energy balance (Caron et al., 2018). These findings therefore show dissociation between the effects of leptin on glucose homeostasis and feeding and body weight regulation. Overexpression of suppressor of cytokine signaling-3 (*Socs3*) in POMC neurons increases adiposity and blocks leptin reduction in food intake (Reed et al., 2010) and POMC deletion of *Socs3* reduces body weight, enhances leptin suppression of food intake, and improves peripheral glucose homeostasis (Kievit et al., 2006). Deletion of the LepR signaling molecule, signal transducer and activator of transcription-3 (*Stat3*), in POMC neurons causes mild obesity in female mice and reduces *Pomc* gene expression (Xu et al., 2007). Consistent with the idea of divergent outputs for leptin signaling in POMC neurons and with the differences between prenatal and adult-specific suppression of leptin signaling, knockdown of *Cacna1e* only in arcuate POMC neurons did not alter food intake, leptin suppression of feeding, or body weight but caused defective regulation of HGP and insulin resistance in adult mice. The elevated fasted serum insulin may have occurred due to hepatic insulin resistance in order to maintain normal glucose levels. In contrast, deletion of *Trpc5* in all POMC neurons leads to an age-dependent increase in body weight, with increased food intake, decreased energy expenditure, and attenuated leptin-mediated anorexia, although glucose homeostasis was unchanged (Gao et al., 2017). Our data indicate a subtle change in insulin sensitivity detected using highly sensitive hyperinsulinemic-euglycemic clamps. It is also possible that reduced resting excitability by *Trpc5* deletion in POMC neurons increases body weight and feeding independent of leptin. Indeed, POMC *Trpc5* expression is required, in part, for effective serotoninergic action on feeding. Moreover, the mildly obese phenotype in *Trpc5*-deleted mice may contribute to leptin resistance attenuating leptin-induced anorexia and excitability.

In summary, the R-type (Ca_V_2.3 or *Cacna1e*) channel is required for long-term leptin depolarization and excitation of POMC neurons *in vitro*. Furthermore, our findings suggest *Cacna1e* is an important component of the ion channel assemblage in arcuate POMC neurons required for the maintenance of normal glucose homeostasis.

## STAR★Methods

### Key Resources Table

**Table:** 

REAGENT or RESOURCE	SOURCE	IDENTIFIER
**Antibodies**		

Ca_V_2.3	Alomone Labs	Cat. ACC-006; RRID:AB_2039777
Na^+^/K^+^-ATPase α-1	Millipore	Cat. 05-369; RRID:AB_309699
Rabbit anti-POMC(27-52)	Phoenix Pharmaceutics Inc.	Cat. H-029-30; RRID:AB_2307442
Chicken anti-rabbit-alex-488	Molecular Probes	Cat. A21441; RRID:AB_141735

**Chemicals, Peptides, and Recombinant Proteins**		

Leptin	R&D Systems	Cat. 498-OB

**Critical Commercial Assays**		

Leptin ELISA	Millipore	Cat. EZML-82K
Insulin ELISA	Crystal Chem	Cat. 90080

**Experimental Models: Cell Lines**		

GT1-7	Pamela Mellon, Uni. of California	N/A

**Experimental Models: Organisms/Strains**		

POMCCre	Greg Barsh. Uni. of Stanford	N/A
NestinCre	Jackson Labs	B6.Cg-Tg(Nes-cre)1Kln/J
Z/EG	Jackson Labs	Tg(CAG-Bgeo/GFP21Lbe/J
POMC-GFP	Jackson Labs	B6.Cg-Tg(Pomc-MAPT/Topaz)1RCK/J

**Oligonucleotides**		

*Gck*	ThermoFisher	Mm00439129_m1
*G6Pc*	ThermoFisher	Mm00839363_m1
*Hprt*	ThermoFisher	Mm00446968_m1
*Pck1*	ThermoFisher	Mm01247058_m1
*Pomc*	ThermoFisher	Mm00435874_m1
*Slc2a2*	ThermoFisher	Mm0044622_m1
*IL6*	ThermoFisher	Mm00446190_m1

**Recombinant DNA**		

pAAV-EFa-DIO-hChR2-mCherry-WPRE	Karl Deisseroth, Uni. of Stanford	N/A
pcDNA3.1 expression vector	ThermoFisher	Cat. V79020
pLK0.1 lentiviral expression vector shRNA (Cacna1e kit)	Thermo Scientific	Cat. RMM4534
pAAV-U6-FloxSTOP-shRNA(Cacna1e)-CMV-mCherry	This paper	N/A
pAM-FLEX-GFP	Bill Wisden, Imperial College London	N/A

**Software and Algorithms**		

pClamp10	Molecular Devices	N/A
IgorPro7.0	Wavemetrics	N/A
Photoshop	Adobe	N/A

### Contact for Reagent and Resource Sharing

Further information and requests for resources and reagents should be directed to the Lead Contact, Professor Dominic J. Withers (d.withers@imperial.ac.uk).

### Experimental Model and Subject Details

#### In-vivo animal studies

POMCCre (Xu et al., 2005), NestinCre (B6.Cg-Tg(Nes-cre)1Kln/J), Z/EG (Tg(CAG-Bgeo/GFP21Lbe/J) and POMCGFP (B6.Cg-Tg(Pomc-MAPT/Topaz)1RCK/J) (Pinto et al., 2004) mice were bred on a C57BL/6J background and maintained on a 12 h light/dark cycle with free access to water and standard mouse chow (4.25% fat, RM3, Special Diet Services). Mice were housed in specific-pathogen free barrier facilities in individually ventilated cages of mixed genotypes. Male transgenic mice were age-matched (4-8 months) and studied with littermate controls. Mice were handled and all studies performed in accordance to the United Kingdom Animals (Scientific Procedures) Act (1986), amended regulations (2012), and approved by Imperial College and University of Cambridge Animal Welfare and Ethical Review Bodies. Findings and experiments described in this paper were designed and reported following the Animal Research: Reporting of *In Vivo* Experiments (ARRIVE) guidelines of animal experiment reporting (Kilkenny et al., 2010). Where possible, investigators were blinded to the genotype of both study animals and that of tissue and blood samples. For experiments involving treatments, mice were randomized by genotype to study groups or a cross-over design was used where indicated and study cohorts were matched for initial bodyweight where appropriate. Treatments were administered in random order. Mice were group housed (3-5 per cage) unless stated.

#### Stereotactic surgery

Male (4-6 month) POMCCre and NestinCre mice were anesthetized with isoflurane and placed in a KOPF stereotaxic frame. Analgesia was administered by topical bupivacaine (8mg/kg) and subcutaneous injection (5mg/kg) of carprofen during surgery, followed by the addition of carprofen to the drinking water (0.0272mg/ml) for 3-5 days post-surgery. Adeno-associated viral particles (AAV1 capsid) containing the cre-dependent shRNA construct (0.6-2.8 × 10^13^ GC/ml) or shRNA scramble (2.0 × 10^13^ GC/ml) were injected bilaterally with a Hamilton syringe (0.3 μl/min, 0.3 μL per inject site) into the arcuate nucleus at −1.2 mm posterior to bregma, ± 0.4 mm lateral to the midline and −6.1, −5.8 mm ventral to the surface of the skull. Co-ordinates for cortical injections into NestinCre mice were +0.5 mm posterior and ± 1.8 mm lateral from bregma and −2.0, −1.5 mm ventral from the surface of the skull. Mice were group housed and left for a minimum of 4-weeks post-surgery to recover before metabolic measurements or electrophysiological studies were performed. Viral expression of mCherry was used to confirm correct stereotaxic placement post-mortem.

#### Cell Culture

Hypothalamic GT1-7 cells (Mellon et al., 1990) were maintained in a high glucose Dulbecco’s Modified Eagle Medium (DMEM, Sigma) supplemented with fetal bovine serum (10% v/v), L-glutamine (8 mM), penicillin/streptomycin (2% v/v) and plated on poly-L-lysine coated flasks or glass coverslips. Cell were transfected 2 days following passage (1:6-1:8 density) with FuGENE (Promega) at a ratio of 2:1 reagent to cDNA. Cells were transfected with cDNA containing *Egfp* in the pcDNA3.1 mammalian expression vector, or with the shRNA sequences in the pLK0.1 lentiviral expression vector. Cells expressing the puromycin selection marker in the pLK0.1 vector were treated with puromycin (5 μg/ml) for 2-3 days. All electrophysiological recordings were performed on GT1-7 cells 4-5 days post passage.

### Method Details

#### Metabolic and food intake studies

Studies were performed in the animal’s home cage unless indicated. Bodyweights from group-housed mice were measured weekly at 9-10am up to 5-6 weeks post-surgery. Fasted tail blood was analyzed for serum leptin (Millipore) and insulin (Crystal Chem) by ELISA 8 weeks post-surgery. For food intake studies, mice were group housed until 4 weeks post-surgery and then singly housed. Mice were allowed to acclimatize for 2 weeks and periodically fasted overnight. Ad-libitum food intake was measured over 3 consecutive days and for 24 h following an overnight fast. Food intake was measured from singly housed mice injected with either vehicle or leptin (i.p. 1.5 mg/kg) at 9am and again at 6pm for 3 consecutive days. Treatments with either vehicle or leptin were crossed-over following a week wash-out period.

#### Hyperinsulinemic-euglycemic clamps studies

Clamps were conducted as previously described (Voshol et al., 2001). Animals were anesthetized by intraperitoneal injection of a combination of 6.25 mg/kg acetylpromazine, 6.25 mg/kg midazolam and 0.31 mg/kg fentanyl. An infusion needle was placed into the tail vein and D-[^3^H] glucose (specific activity: 10-20Ci (370-740GBq)/mmol) was infused at a rate of 0.006 MBq/min for 80 min to achieve steady-state levels. Thereafter, insulin (Actrapid; Novo Nordisk) was infused at a constant rate of 0.11 mU/min after a bolus dose of 3.3 mU and D-[^3^H]-glucose was continued at a rate of 0.006 MBq/min. A variable infusion of 12.5% D-glucose was used to maintain blood glucose at euglycemic (basal) levels. Blood glucose was measured with an AlphaTRAK glucometer (Abbott Animal Health) every 5-10 minutes and glucose infusion adjusted accordingly. After 60 minutes from the start of the insulin infusion, ^14^C-2-Deoxy-glucose-phosphate (Specific Activity: 250-350mCi (9.25- 13.0GBq)/mmol) was administered i.v. to assess tissue-specific glucose uptake. Steady-state was reached after 60 minutes and blood samples were taken at 10 minutes intervals over 30 minutes to determine steady-state levels of [^3^H]-glucose. Mice were then killed by cervical dislocation and the organs removed and frozen. To measure plasma [^3^H]-glucose, proteins were precipitated with trichloroacetic acid (final concentration 10%), centrifuged, and supernatant dried and re-suspended in water. The samples were counted using scintillation counting (Hidex Scintilation counter, LabLogic). Muscle and brown adipose tissue samples were homogenized (∼5%–10% wet wt/vol, depending on tissue) in 0.5% percholic acid, centrifuged, supernatants neutralized, and ^14^C-2-Deoxy-glucose-phosphate precipitated using BaOH/ZnSO_4_. Total and precipitated counts of supernatants were subtracted and plasma ^14^C-2-Deoxy-glucosephosphate counts were used to calculate tissue specific uptake. Protein content in homogenates was performed using DC protein assay (BioRad). For subcutaneous and epidermal white adipose tissue, ^14^C-2-Deoxy-glucose-phosphate was extracted using anion exchange columns as described previously (Kim, 2009). The glucose turnover rate (μmol/min) was calculated during the basal period and under steady-state clamp conditions as the rate of tracer infusion (dpm/min) divided by the plasma specific activity of [^3^H] glucose (dpm/μmol). Hyperinsulinemic hepatic glucose production was calculated as the difference between the tracer-derived rate of glucose appearance and the glucose infusion rate.

#### Plasmids

*Egfp* was sub-cloned into the pcDNA3.1 expression vector. Four separate shRNA clones directed toward *Cacna1e* (#1: TRCN0000068910, #2: TRCN0000068912, #3 TRCN0000068908, #4: TRCN0000068911) were purchased from Thermo-Scientific (RMM4534) in the pLK0.1 lentiviral expression vector. A cDNA sequence comprising the U6 promoter, followed by a LoxP flanked stop cassette (Hitz et al., 2007) and the shRNA sequence (#2: TRCN0000068912) was synthesized by GeneArt (Life Technologies). Unique restriction sites *Spe1* and *Swa1* were inserted at the ‘3 end of the synthesized cDNA. The cDNA was subcloned into a pAAV expression vector (pAAV-EFa-DIO-hChR2-mCherry-WPRE) between *Mlu1* and *EcoR1* removing the *EFa-DIO-hChR2-mCherry* sequence from the plasmid at the same time. A sequence containing the CMV promoter and mCherry was then synthesized (GeneArt) and inserted into the pAAV vector using the *Spe1* and *Swa1* sites to make the final construct (pAAV-U6-[LoxP-STOP-LoxP]-shRNA[*Cacna1e*]-CMV-mCherry-WPRE). Scramble shRNA (GAGAATACCGACAAAGATACT, designed using Invivogen’s siRNA Wizard software) was synthesized by GeneArt (Life Technologies) and inserted into a pAM-FLEX-GFP vector (Murray et al., 2011).

#### Genotyping

Generic Cre-recombinase (forward: 5′-AGCGATGGATTTCCGTCTCT and reverse: 5′-CACCAGCTTGCATGATCTCC) and GFP (forward: 5′-AGCTAGCCACCATGGTGAGCAAGG GCGAGGAG and reverse: 5′-ATCTCGAGCTTGTACAGCTCGTCCATGCCG) primers were used to genotype POMCCre, NestinCre and POMCGFP mice. Positive cre-recombinase bands were observed at approximately 200 bp and for GFP at 600 bp.

#### Quantitative RT-PCR analysis

Tissues were lysed and homogenized in TRIzol reagent (Ambion) and total RNA was isolated using the RNeasy mini kit (QIAGEN). First-strand cDNA was generated using Taqman reverse transcription reagents (Applied Biosystems) and qPCR was performed using Taqman universal PCR mastermix in a 7900HT real-time PCR system (Applied Biosystems). mRNA quantities were normalized to *Hprt* after determination by the comparative Ct method. Primers used were: *Gck* (Mm00439129_m1), *G6Pc* (Mm00839363_m1) *Hprt* (Mm00446968_m1), *Pck1* (Mm01247058_m1), *Pomc* (Mm00435874_m1), *Slc2a2* (Mm0044622_m1), *IL6* (Mm00446190_m1).

#### Western blot analysis

Cortices were removed and homogenized in lysis buffer (50 mM Tris pH 7.4, 150 mM NaCl, 1 mM EDTA, 1% w/v Triton X-100) supplemented with Roche complete protease inhibitor cocktail and phosphatase inhibitors (1 mM sodium orthovadanate, 5 mM sodium fluoride and 2 mM β-glycerophosphate). 20-100 μg of total protein homogenates were run on 15% SDS-PAGE gels, transferred to nitrocellulose membranes and blotted with antibodies against Ca_V_2.3 (1:1000, #ACC-006 Alomone Labs) and Na^+^/K^+^-ATPase α-1 (1:1000, #05-369 Millipore). Detection was performed using enhanced chemiluminescence (Luminata Crescento, Millipore) and exposed on films. The intensity of the bands was quantified by densitometry using Adobe Photoshop software and normalized to Na^+^/K^+^-ATPase protein.

#### Hypothalamic immunohistochemistry

Mice were perfused with paraformaldehyde (4% w/v) and frozen coronal sections (35 μm) were cut for immunohistochemistry as previously described (Choudhury et al., 2005). Arcuate sections were incubated with rabbit anti-POMC precursor (1:1000; Phoenix Pharmaceuticals Inc.) and detection performed using a secondary antibody coupled to Alexa Fluor-488 (1:200, Molecular Probes).

#### Electrophysiology

Mice were killed by cervical dislocation and hypothalamic coronal slices (350 μm) were cut from aged matched (5-6 month old) transgenic mice expressing POMCCre and POMC-GFP or POMCCre and Z/EG. Slices were maintained at room temperature (22-25°C) in an external solution containing (in mM) NaCl 125, KCl 2.5, NaH_2_PO_4_ 1.25, NaHCO_3_ 25, CaCl_2_ 2, MgCl_2_ 1, D-glucose 10, D-mannitol 15, equilibrated with 95% O_2_, 5% CO_2_, pH 7.4. For intact slice studies, POMC neurons were visualized in the arcuate nucleus by video-enhanced differential interference contrast microscopy and by the expression and excitation of GFP. In addition, arcuate wedges from slices containing GFP labeled POMC neurons were dissected and incubated for 1 h in the standard external solution containing 0.5 mg/ml protease (type XIV) at room temperature. Following several washes with a HEPES (10 mM, pH 7.4) based external solution, individual neurons were dispersed by gentle trituration with fire polished glass Pasteur pipettes with decreasing tip aperture, as previously described (Spanswick et al., 1997). The cell suspension was evenly plated on concanavalin A (1 mg/ml) coated 35 mm culture dishes (Falcon) and left for 1 hour at 4°C allowing cell adhesion prior to use. Cells were visualized by phase contrast microscopy and POMC neurons identified by GFP expression and excitation.

Whole-cell current-clamp recordings were made at 33°C using borosilicate glass pipettes (4-8 MΩ) containing (in mM) Kgluconate 130, KCl 10, EGTA 0.5, NaCl 1, CaCl_2_ 0.28, MgCl_2_ 3, Na_2_ATP 3, GTP 0.3, phosphocreatine 14 and HEPES 10 (pH 7.2). Following the establishment of a stable recording, leptin (R&D Systems) was applied for 2 minutes using a broken tipped pipette (∼3 μm) positioned above the recording neuron. For qualitative descriptions only, responsive neurons were distinguished from non-responding neurons based on the criterion that the change in membrane potential by leptin challenge was ± three times the standard deviation prior to addition of the drug. In voltage-clamp studies, neurons were recorded in the standard external solution in the presence of 1 μM tetrodotoxin, 20 μM (+)-bicuculline and 2,3-dihydroxy-6-nitro-7-sulfamoyl-benzo(f)quinoxaline-2,3-dione (NBQX, 5 μM) with D-2-amino-5-phosphonopentanoate (D-AP5, 50 μM). Neurons that changed series resistance (10-30 MΩ) were excluded from the analysis. Neurons were held at −70 mV and voltage-ramped (−10 to −100 mV over 2 s) every minute. In order to identify the conductances activated by leptin, the mean of 5 voltage ramps before leptin application was subtracted from mean of 5 ramps following leptin application. Voltage-gated calcium (Ca_V_) currents were recorded with a standard internal solution but with K^+^-gluconate replaced by 130 mM CsCl. The Ca_V_ conductance was further isolated by an external solution containing in mM: NaCl 125, KCl 2.5, BaCl_2_ 10, D-glucose 10, D-mannitol 15 and HEPES 10, pH 7.4. Series resistance and whole-cell capacitance were compensated (40%–70%) using an Axopatch 200B amplifier. Any uncompensated resistance and capacitance were digitally (IgorPro7) subtracted using an equivalent voltage-step protocol in which Ca_V_ was inactivated by voltage-clamping the neuron at 0 mV for ≥ 2 minutes. Leptin was applied for 2 minutes following the establishment of a stable recording. An average of 5 consecutive trials before and immediately after leptin application was used to assess mean change in current.

Stock chemicals were dissolved and added to the appropriate external or internal solution prior to recording. Tetrodotoxin, SNX-482 and D-AP5 were from Tocris (UK); ω-conotoxin GVIA and ω-agatoxin IVA were purchased from Alomone Labs (Israel); mibefradil, (+)-bicuculline, NBQX and nimodipine were from Sigma-Aldrich

### Quantification and Statistical Analysis

Statistical significance was calculated from all neurons (responsive and non-responsive) using repeated-measures (RM) two-way ANOVA and one-way ANOVA followed by Bonferroni post hoc analysis, Student’s two-tailed paired and unpaired t test, or one-sample t test where stated. Data are expressed as mean ± standard error of mean and degrees of freedom shown in brackets. A maximum of 2 recordings were made from any given mouse. Recordings and observations were repeated on at least 4 different mice. Study cohort sizes were determined by power calculations based on our previous data in mice with targeted hypothalamic mutations.

## Supplemental Information

Document S1. Figure S1

Document S2. Article plus Supplemental Information
