# Supplementary material for: Calcium Channel CaV2.3 Subunits Regulate Hepatic Glucose Production by Modulating Leptin-Induced Excitation of Arcuate Pro-opiomelanocortin Neurons
Source: Cell Rep. 2018 Oct 9;25(2):278–287.e4. doi: 10.1016/j.celrep.2018.09.024 (PMC6198286; doi:10.1016/j.celrep.2018.09.024)
Supplement: Document S1. Figure S1 [file mmc1.pdf]

Cell Reports, Volume *25*

Supplemental Information

Calcium Channel $Ca_V2.3$ Subunits Regulate Hepatic Glucose Production by Modulating Leptin-Induced Excitation of Arcuate Pro-opiomelanocortin Neurons

Mark A. Smith, Loukia Katsouri, Samuel Virtue, Agharul I. Choudhury, Antonio Vidal-Puig, Michael L.J. Ashford, and Dominic J. Withers

SUPPLEMENTAL INFORMATION

FIGURE S1

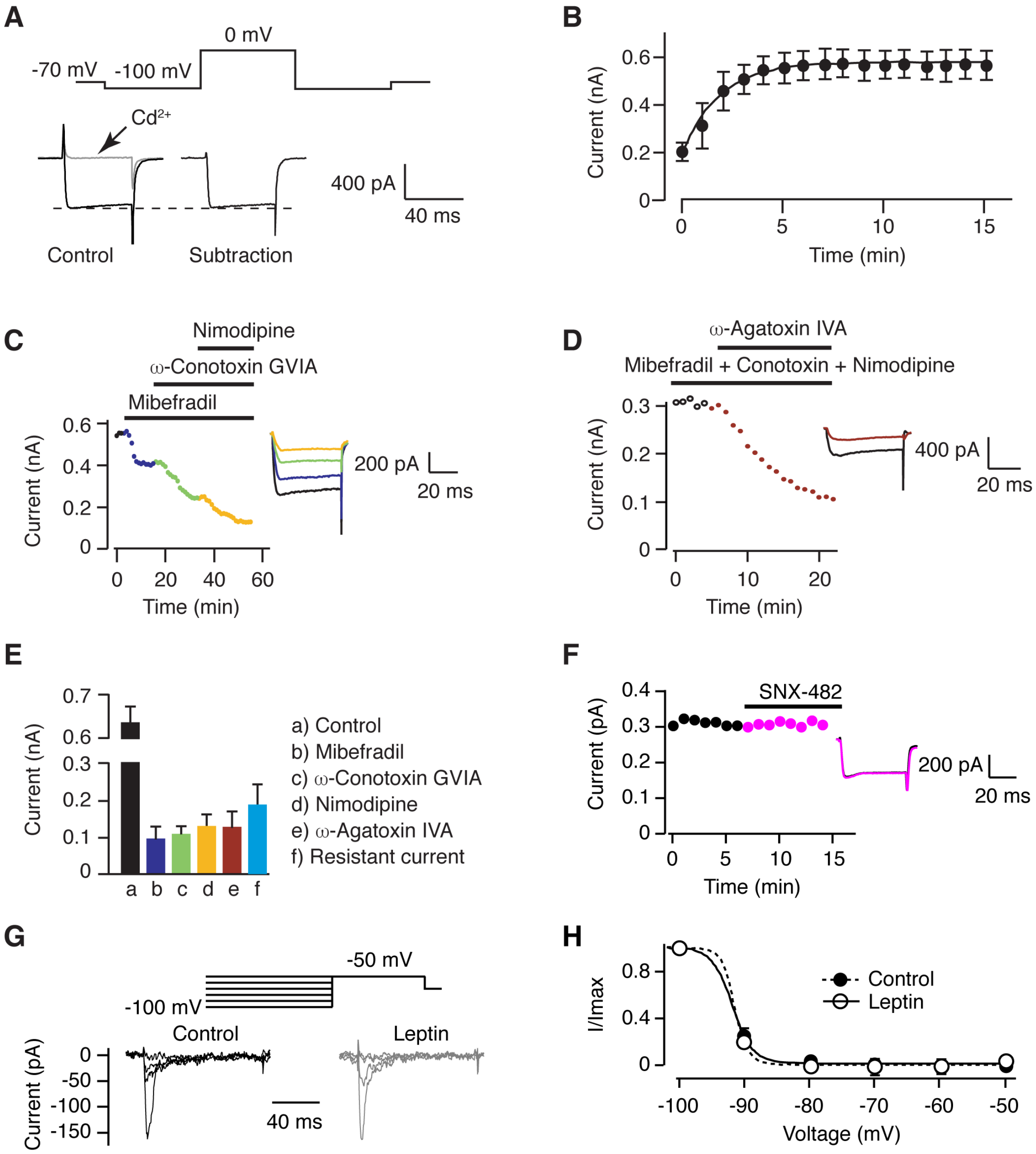

Figure S1. Pharmacological profile of the voltage-dependent barium current of POMC neurons (related to Figure 1).

(A) Representative Ba^{2+} currents in the presence and absence of $CdCl_2$ (100 μM, where indicated) evoked by voltage-steps (-100 to 0 mV, 50 ms duration) from POMC neurons in intact slices

(B) Diary plot of barium current against time following the attainment of the whole-cell configuration (mean ± SEM, n = 10).

(C) Ba^{2+} currents were evoked in the presence and absence of selective calcium channel antagonists, as denoted by the diary plots (left) and examples of steady-state currents (right). Control (black) currents were partially blocked by mibefradil (10 μM, dark blue), ω-conotoxin GVIA (200 nM, green) and nimodipine (10 μM, yellow).

(D) In the constant presence of mibefradil, ω-conotoxin GVIA and nimodipine (open circles and black corresponding trace), ω-agatoxin IVA (200 nM, red) further reduced the Ba^{2+} current amplitude, although a current, resistant to this cocktail of blockers, remained.

(E) Bar chart, showing the mean magnitude of Ba^{2+} current in the absence of blockers (a), sensitive to inhibition by the various calcium channel antagonists (b - e) and resistant to block (f), (mean ± SEM, n = 5 - 7).

(F) Representative diary plot (left) and current trace (right) of Ba^{2+} currents evoked at 0 mV in the presence and absence of SNX-482 (50 nM).

(G) Representative Ba^{2+} currents from dispersed POMC neurons evoked at –50 mV following a 5 s duration steady-state inactivation protocol (–100 to –50 mV in 10 mV steps). Currents are shown in the absence (black) and presence (grey) of leptin (50 nM).

(H) Steady-state inactivation for currents evoked by the protocol described in (I), in the absence (closed circles) and presence (open circles) of leptin. Data points (mean ± SEM, n = 4) were best fit with the Boltzmann equation.
